# Involvement of heat shock proteins HSP70 in the mechanisms of endogenous neuroprotection: the prospect of using HSP70 modulators

**DOI:** 10.3389/fncel.2023.1131683

**Published:** 2023-04-17

**Authors:** Igor F. Belenichev, Olena G. Aliyeva, Olena O. Popazova, Nina V. Bukhtiyarova

**Affiliations:** ^1^Department of Pharmacology and Medical Formulation With Course of Normal Physiology, Zaporizhzhia State Medical University, Zaporizhzhia, Ukraine; ^2^Department of Medical Biology, Parasitology and Genetics, Zaporizhzhia State Medical University, Zaporizhzhia, Ukraine; ^3^Department of Histology, Cytology and Embryology, Zaporizhzhia State Medical University, Zaporizhzhia, Ukraine; ^4^Department of Clinical Laboratory Diagnostics, Zaporizhzhia State Medical University, Zaporizhzhia, Ukraine

**Keywords:** heat shock proteins, CNS, ischemia, hypoxia, neuroprotection, HIF-1a, HSP70 modulators

## Abstract

This analytical review summarizes literature data and our own research on HSP70-dependent mechanisms of neuroprotection and discusses potential pharmacological agents that can influence HSP70 expression to improve neurological outcomes and effective therapy. The authors formed a systemic concepts of the role of HSP70-dependent mechanisms of endogenous neuroprotection aimed at stopping the formation of mitochondrial dysfunction, activation of apoptosis, desensitization of estrogen receptors, reduction of oxidative and nitrosative stress, prevention of morpho-functional changes in brain cells during cerebral ischemia, and experimentally substantiated new target links for neuroprotection. Heat shock proteins (HSPs) are an evolutionarily integral part of the functioning of all cells acting as intracellular chaperones that support cell proteostasis under normal and various stress conditions (hyperthermia, hypoxia, oxidative stress, radiation, etc.). The greatest curiosity in conditions of ischemic brain damage is the HSP70 protein, as an important component of the endogenous neuroprotection system, which, first of all, performs the function of intracellular chaperones and ensures the processes of folding, holding and transport of synthesized proteins, as well as their degradation, both under normoxic conditions and stress-induced denaturation. A direct neuroprotective effect of HSP70 has been established, which is realized through the regulation the processes of apoptosis and cell necrosis due to a long-term effect on the synthesis of antioxidant enzymes, chaperone activity, and stabilization of active enzymes. An increase in the level of HSP70 leads to the normalization of the glutathione link of the thiol-disulfide system and an increase in the resistance of cells to ischemia. HSP 70 is able to activate and regulate compensatory ATP synthesis pathways during ischemia. It was found that in response to the cerebral ischemia formation, HIF-1a is expressed, which initiates the launch of compensatory mechanisms for energy production. Subsequently, the regulation of these processes switches to HSP70, which “prolongs” the action of HIF-1a, and also independently maintains the expression of mitochondrial NAD-dependent malate dehydrogenase activity, thereby maintaining the activity of the malate-aspartate shuttle mechanism for a long time. During ischemia of organs and tissues, HSP70 performs a protective function, which is realized through increased synthesis of antioxidant enzymes, stabilization of oxidatively damaged macromolecules, and direct anti-apoptotic and mitoprotective action. Such a role of these proteins in cellular reactions during ischemia raises the question of the development of new neuroprotective agents which are able to provide modulation/protection of the genes encoding the synthesis of HSP 70 and HIF-1a proteins. Numerous studies of recent years have noted the important role of HSP70 in the implementation of the mechanisms of metabolic adaptation, neuroplasticity and neuroprotection of brain cells, so the positive modulation of the HSP70 system is a perspective concept of neuroprotection, which can improve the efficiency of the treatment of ischemic-hypoxic brain damage and be the basis for substantiating of the feasibility of using of HSP70 modulators as promising neuroprotectors.

## 1. Introduction

Currently, a significant amount of information has been accumulated on almost all links of the pathological process triggered by ischemic damage, however, there are significant gaps regarding the mechanisms of endogenous neuroprotection that require further study (Belenichev et al., 2015a; Sheng et al., 2018; Kim et al., 2020a).

It is known that the response to any damage in the body is the activation of its own system of cytoprotection. Thus, ischemic circulatory disorders in the brain lead to the development of hypoxic stress and the launch of adaptive mechanisms of endogenous neuroprotection. Numerous studies have established a large number of intracellular signaling molecules that affect the survival of neurons after ischemia, among them: tumor necrosis factor-α (TNF-α), nuclear factor kappa B (NF-κB), hypoxia-inducible factor 1 (HIF-1), inducible nitric oxide synthase (iNOS), etc. (Ginsberg, 2016).

One of the most studied cytoprotective factors is the HSPs (heat shock proteins) family (Kim et al., 2019). One of the primary reactions of the genome in response to stress of various origins is the induction of HSPs, which are named according to their molecular weights. There is a class of proteins (chaperones), whose main function is to restore the correct tertiary structure of damaged proteins, as well as the formation and dissociation of protein complexes. Many chaperones are HSPs, that is, proteins whose expression begins in response to an increase in temperature or other cellular stresses (Deka and Saha, 2018; Kaur and Asea, 2019; Kim et al., 2019, 2020a).

Heat shock proteins are an evolutionarily integral part of the functioning of all cells, as evidenced by their high degree of homology and presence in all existing organisms (Clerico et al., 2019).

Heat shock proteins were first detected in *Drosophila melanogaster* as a result of incubation at elevated temperatures. Therefore, the synthesis of these proteins was originally considered a temperature-dependent process. Gradually, in many studies, an increase in the level and expression of HSPs was noted in various experimental stress models that did not depend on temperature conditions (UV irradiation, heavy metals, inflammation, ischemia, etc.) (Kampinga and Craig, 2010; Rosenzweig et al., 2019; Zhang et al., 2022).

Heat shock proteins act as intracellular chaperones that support cell proteostasis under normal and various stress conditions (hyperthermia, hypoxia, oxidative stress, radiation, etc.). According to the molecular weight HSPs are usually classified into six subfamilies: HSPH (HSP110), HSPC (HSP90), HSPA (HSP70), DNAJ (HSP40), HSPB [small HSPs (sHSPs)], and the chaperonin families: HSPD/E (HSP60/HSP10) and CCT [cytosolic chaperonin TCP1 ring complex (TRIC)]. Although the overall task of these proteins in maintaining cellular life remains common, the function and tissue specificity of HSPs differ between groups under physiological and stress conditions (Deka and Saha, 2018; Kim et al., 2019).

Eukaryotes often express several members of the HSP70 family, the main isoforms of which are found in all cellular compartments: Hsp72 (HSPA1A) and related HSP70 (Hsc70/HSPA8), in the cytosol and nucleus, BiP (Grp78/HSPA5) in the endoplasmic reticulum, and mtHSP70 (Grp75/mortalin/HSPA9) in mitochondria. HSP70, as an important component of the endogenous neuroprotection system, is the greatest curiosity in conditions of ischemic brain damage. Hsp70 performs the function of intracellular chaperon and plays an important role in the protection of cells from stress, by binding to unfolded proteins and preventing them from aggregating. Additionally, Hsp70 is involved in the folding, holding and transport of newly synthesized proteins, and in the repair of damaged proteins. Hsp70 is also involved in the regulation of gene expression, and in the regulation of protein degradation (Giffard and Yenari, 2004; Kim et al., 2018; Rosenzweig et al., 2019). The cytosolic isoforms, HSP70 and HSP72, are partially functionally redundant, but HSP72 transcription is highly sensitive to stress, and cytosolic HSP70 is constitutively expressed (Nitika et al., 2020). In the endoplasmic reticulum and mitochondria, members of the HSP70 family are believed to perform specific functions and have unique substrates, while BiP plays a key role in folding and quality control of proteins in the endoplasmic reticulum, and mtHSP70 takes part in the import and export of proteins from mitochondria (Evans et al., 2010). Such a role of these proteins in cellular reactions during ischemia raises the question of the development of new neuroprotective agents which are able to provide modulation/protection of the genes encoding the synthesis of HSP70 proteins. HSP70 plays a protective role in damage to the nervous system, it acts as a chaperone and protects neurons from protein aggregation and excitotoxicity, inhibits neuroapoptosis, neuroinflammation, and exhibits an antioxidant effect. A number of studies have shown the neuroprotective effects of HSP70 caused by an increase in its expression by drugs. These studies substantiate brain protection strategies during ischemia and neurodegeneration.

## 2. The role of HSP70 proteins in the mechanisms of endogenous neuroprotection

It is known that the family of HSP70 includes: inducible/stress block HSP72/HSP70i, constitutive/physiological protein HSP73/HSP70, glucose-regulated protein 78 (GRP78) and GRP75, which plays a critical role in maintenance of cellular homeostasis, constitutive endoplasmic protein GRP73, and inducible heme oxygenase-1 (HO-1) involved in bilirubin metabolism (Kim et al., 2020a; Demyanenko et al., 2021). The constitutive form of HSP70 is equally present in all subcellular compartments and is involved in the operation of cell life support systems under normoxic conditions. On the contrary, the inducible form of HSP70 appears in cells in response to stress, including during ischemic stroke (Belenichev and Litvinenko, 2017; Pavlov et al., 2017a; Craig, 2018; Dukay et al., 2019; Rosenzweig et al., 2019).

First of all, HSP70 is considered as a chaperone protein that provides powerful cell proteostasis. The chaperone function of HSP70 is to interact with damaged, denatured, with subsequent determination of their fraction (Clerico et al., 2019). The appearance and accumulation of damaged proteins in the cell increases the expression of HSP70. After thermal injury causing denaturation of all proteins, the concentration of HSP70 can be up to 20% of all cytoplasmic proteins. The action of HSP70 leads to the formation of thermotolerance – the phenomenon of adaptation to elevated temperatures and an increase in the temperature threshold of sensitivity due to the stabilization of protein molecules with HSP70 during initial, less intense heating (Ramos et al., 2017). HSP70 proteins are necessary for cell repair and survival under stressful conditions, their amount correlates with the severity of various pathological conditions, such as ischemia, inflammation, autoimmune processes, malignant tumors, bacterial and viral infections, and neurodegenerative diseases (Ortan et al., 2018; Kim et al., 2020b). The participation of HSP70 in the mechanisms of holding, folding, and refolding of improperly assembled proteins, as well as the disaggregation and elimination of denatured proteins accumulating under stress conditions, protects the membranes of organelles, primarily mitochondria, and the cells themselves from damage (Aufricht, 2005; Belenichev, 2013; Clerico et al., 2015; Mayer and Gierasch., 2019).

**FIGURE 1:**
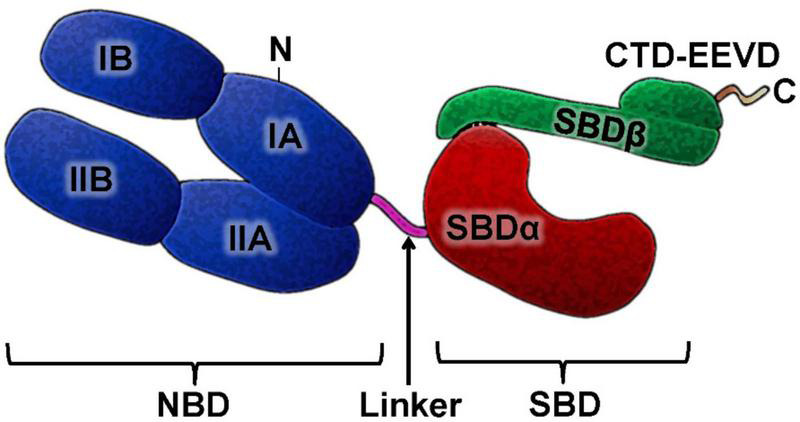
HSP70 structure. The HSP70 family members has a highly conserved N-terminal nucleotide-binding domain (NBD) and C-terminal substrate-binding domain SBD and in its structure. NBD and SBD are connected by a linker. The SBD domain is subdivided into a sandwich subdomain (SBDα) and α-helical lid subdomain (SBDβ) followed by unstructured C-terminal domain (CTD), which in many cytosolic Hsp70s is often ends with a charged EEVD motif.

The HSP70 family is known to be very conserved during evolution. The molecular structure of HSP70 has two functional domains: an N-terminal nucleotide binding domain (NBD) and a C-terminal substrate binding domain (SBD), which are connected by a linker. The NBD is subdivided into four subdomains (IA, IB in the lobe I and IIA, IIB in the lobe II), and there is a gap between the two lobes, which is the ATP binding site. SBD is subdivided into a β-sandwich subdomain (SBDβ) and an α-helical subdomain (SBDα), followed by a variable length C-terminal tail [C-terminal domain (CTD)]. The N-terminal NBD provides an ATP/ADP pocket for ATP binding, which is critical for the ATPase reaction necessary for folding and release of proteins. The SBD domain is a pocket for peptide binding (Figure 1).

To maintain the chaperone activity of HSP70, the presence of co-chaperones HSP40 and HSP10, which are hydrophobic regions of proteins and regulate the ATP/ADP exchange, is necessary. The HSP70/HSP40 complex ensures the *de novo* assembly of the synthesized protein chain, its transportation into cell organelles and subsequent refolding, preventing interprotein aggregation, maintaining the activity of polypeptides and the degradation of proteins that cannot be restored (Belenichev, 2013; Alderson et al., 2014; Ortan et al., 2018; Mayer and Gierasch, 2019; Kim et al., 2020a).

In response to ischemia, a sharp increase in the level of HSP70 is recorded, while its highest concentration is observed in vital parts of cells: nuclear, perinuclear space, mitochondria, and endoplasmic reticulum, which indicates the importance of chaperone 70 in protection against death (Sharp et al., 2013). As the work of nuclear preribosomes resumes, the concentration of HSP70 in the nucleus decreases and increases in the cytoplasm of cells. Thus, the HSP70 level can be considered as a marker of cellular and tissue damage. Hyperproduction of HSP70 in cells inhibits the development of autophagy as an alternative, more “radical” mechanism of cellular response to stress (Giffard and Yenari, 2004; Sharp et al., 2013; Paul and Mahanta, 2014; Leu et al., 2017; Dukay et al., 2019).

Scientific works of recent years have established a direct cytoprotective effect of HSP70, which is implemented through the regulation of apoptosis and cell necrosis. HSP70 inhibits the mitochondrial and cytoplasmic pathways of apoptosis. Thus, HSP70 inhibits the transition of procaspase 9 to active caspase 9 and disrupts the formation of apoptosome in the cell cytoplasm. During HSP70 overexpression, the level of the anti-apoptotic protein Bcl-2 increases, which prevents the release of cytochrome c from mitochondria and the translocation of the apoptosis-inducing factor (AIF) into the nucleus, which prevents cell apoptosis. The HSP70 protein inhibits TNFα-induced apoptosis and also effectively inhibits the development of Fas- and TRAIL (TNF-related apoptosis-inducing ligand) mediated apoptosis in various cell types. The accumulation of HSP70 in cells increases the resistance of the latter to staurosporine and doxorubicin, known inducers of apoptosis (Naka et al., 2014).

It is known that ischemia leads to the development of local inflammation, which increases the area of brain damage. In this situation, HSP70 blocks the activation of the inflammatory transcription factor NF-κB and suppresses its cytokine-mediated translocation to the nucleus (Jo et al., 2006). In *in vitro* and *in vivo* models of ischemia, HSP70 inhibits the production of pro-inflammatory cytokines (TNF-α and IL-6), inhibits the activity of matrix metalloprotein kinases (MMPs) and iNOS (Lee et al., 2004; Dukay et al., 2019). It has been established that HSP70 in the culture of astrocytes against during ischemia inhibits proapoptotic Jun N-terminal kinases (JNK) and p38 mitogen-activating protein kinase (MAPK), disrupting the signaling pathway of apoptosis (Wang et al., 2012). The works show a direct relationship between the level of expression of HSP70 and TNFα, iNOS in microglia and MMP-9 in astrocytes. Accordingly, this is another mechanism of action of HSP70 aimed at reducing the size of ischemic damage due to degradation of the extracellular matrix (Barreto et al., 2012; Lee et al., 2012; Leu et al., 2017; Kim et al., 2018).

An important role of HSP70 in the activation of endogenous evolutionarily acquired genetically determined brain defense mechanisms has been established in numerous models of ischemic pre- and postconditioning. It has been established that short-term ischemia induces the synthesis of HSP70 and significantly increases the resistance of brain and myocardial tissues to the next long-term ischemia (Nagai et al., 2010; Sakakima, 2019; Song et al., 2019; Wang et al., 2019).

Various types of cellular stress can lead not only to an increase in the intracellular content of HSP70. In some cases, stress induces HSP70 translocation to the plasma membrane and/or release of these proteins into the extracellular space, which leads to the formation of an extracellular pool of these proteins freely circulating in the body. For the first time, the release of HSP into an extracellular site was recorded in the study of nerve cells. It has been proven that HSP can be released from glial cells (Paul and Mahanta, 2014; Belenichev et al., 2015a; Kim et al., 2018).

Extracellular HSP70 plays the role of a mediator in the processes of intercellular interactions and protein transport. After HSP70 is released into the extracellular space, it can bind to the cell surface via an autocrine or paracrine mechanism. An alternative mechanism of cellular interaction involving HSP70 may be associated with membrane vesicles containing HSP70. These vesicles can be released from cells and then taken up by other cells through membrane fusion or through receptor interaction. One of the first works on this topic described that after the release of HSP70 from glia into the extracellular space, it is taken up by surrounding neurons in the axonal region. The diversity of receptors that interact with HSP70 or are indirectly activated by HSP70 indicates that these proteins interact with different cell types and perform a number of functions outside the cell (Tukaj, 2020).

In recent years, much information has emerged about the importance of extracellular HSP70 in the regulation of the inflammatory process that occurs in response to injury. As a result of cell death, HSP70 is released into the extracellular space and acts as a signal “danger molecule” by interacting with TLR2 and TLR4 receptors of macrophages, glial, and dendritic cells. Interaction with Toll-like receptors leads to the activation of NF-κB, resulting in the activation of the expression of pro-inflammatory cytokines and iNOS (Zheng et al., 2008; Sun et al., 2015; Ortan et al., 2018; Dukay et al., 2019).

In many research papers devoted to the study of the mechanisms of endogenous neuroprotection under conditions of ischemia, the resumption of the work of the glutathione unit of the thiol-disulfide system (TDS) during an increase in the level of HSP70 is noted, and the administration of exogenous HSP70 leads to an increase in the functional activity of the glutathione system in ischemic neurons of experimental animals (Hacke et al., 2008). Thus, under conditions of ischemia, HSP70 proteins mobilize antioxidant resources in neurons by increasing the level of both the cytosolic and mitochondrial pool of reduced glutathione (Lener et al., 2015). Recently, data have appeared on the role of HSP in the stabilization of HIF-1a, which, under conditions of ischemia, is responsible for ensuring the processes of proliferation, apoptosis, angiogenesis, and stabilization of protein molecules under conditions of oxidative stress. It was also shown that under conditions of hypoxia, HSP70 is displaced from the complex with HIF-1a, which, during 20–30 min of hypoxia, protects the structure of the factor from targeted proteolysis. It is likely that HSP70 is able to increase the lifetime of the HIF-1a factor under conditions before and after hypoxia and is necessary for cells to properly respond to oxygen starvation in conditions of acute cerebrovascular accident (Jo et al., 2006; Kadhim et al., 2008; Kampinga and Craig, 2010; Kityk et al., 2015; Kim et al., 2018, 2020a). Activation of microglial products in the penumbra leads to transient activation of genes encoding transcription factors (including c-Fos) in the first few minutes from the onset of a stroke. Then the second wave of expression of HSP genes (HSP70 and HSP72) starts, increasing during the first 1–2 h of the disease and decreasing on the first and second day (Jo et al., 2006; Shulyatnikova and Shavrin, 2012; Hernandez-Ontiveros et al., 2013).

It was found that in suspensions of neurons with the introduction of glutamate (100 μM) and CDNB (80 μM), an increase in the HSP70 concentration was observed with a maximum increase at 30 min of incubation and a subsequent decrease in concentration by 60 min relative to the initial level (Pavlov et al., 2017a). A significant increase in the HSP70 concentration in the cortex and hippocampus was found on the fourth day after occlusion of the common carotid arteries in animals with mild neurological disorders (according to P. McGraw) compared with the control group (non-operated animals). In the experimental group with a moderate level of neurological deficit, a multidirectional change in the concentration of HSP70 occurred – in the cortex, the content of HSP70 was higher than in the control group, while in the hippocampus, the HSP70 concentration was reduced compared to the control group. The minimum level of HSP70 was observed in the hippocampus of the experimental group with severe neurological symptoms. In the cortex of this group, the level of HSP70 did not differ or was lower than in the control group (Belenichev et al., 2017).

Determination of the HSP70 concentration in the hippocampus at different times after occlusion of the carotid arteries showed that after 12 h a significant increase in protein concentration occurred, and after 24 h the concentration decreased, reaching a maximum by day 4 of the experiment. After 18–21 days of occlusion, the concentration of HSP70 was lower than in the control. In the cerebral cortex of animals after occlusion of the carotid arteries, the concentration of HSP70 was higher than in the control group after 12 h and after 1 day, after 4 days the protein concentration decreased but was at the level or higher than in the control group (Belenichev et al., 2017).

Experimental studies for different periods of ischemia (1 h, 6 h, 24 h, 48 h, 72 h, 120 h, and 21 days) found that during the period of the greatest ischemic damages 24–72 h, hyperproduction of lactate is observed, during the inhibition of GK – enzyme that catalyzes the first reaction of glycolysis. When assessing the dynamics of changes in the oxidative potential, a sharp inhibition of succinate dehydrogenase (SDH) (77–85%) and isocitrate (56–70%) can be noted. The restoration of these indicators begins only by the 21st day of the experiment. Attention is drawn to the initial (from the 1st to the 24th hour) increase in the activity of mitochondrial malate dehydrogenase (MDH) and cytoplasmic MDH with an increase in the level of malate (20–50%), and later (48th–72nd hour) there is a moderate reduction of its activity (10%) with a decrease in the content of malate (16–38%). Thus, we observe a pronounced inhibition of the tricarboxylic acid cycle at the citrate-succinate site. This drastic inhibition of SDH activity makes the implementation of the succinate oxidase pathway for the supply of protons to the respiratory chain problematic. The rise of malate with an increase in the activity of mMDH and cMDH in the first hours of cerebral ischemia indicates the activation of the malate-aspartate shuttle mechanism for the transport of reduced equivalents into mitochondria. The study of bioenergy indicators in the acute period of ischemia (up to 24 h) revealed the following interesting patterns: the most significant changes were in such indicators as the activity of mitochondrial and cytosolic NAD-MDH and NADP-MDH, as well as the content of HSP70 and HIF-1a. A positive correlation was established between changes in the level of malate, NAD-MDH and HSP70 [*m*_*r*_ = 0.821; T-2.94 (m_*r*_ is the multiple correlation coefficient; the significance of this coefficient was estimated by the value of Student’s *t*-test with the number of degrees of freedom *k* = n − 3)]. It was also found that the general trend toward a decrease in malate was associated with the restoration of NADP-MDH and HIF-1a (*m*_*r*_ = 0.839; T-3.09). Mathematical analysis established a direct relationship between the concentration of the HSP70 protein and the level of MDH activity. The study showed that in animals with high scores according to P. McGrow (severe neurological symptoms), the content of malate and HSP70, HIF-1a is the lowest. In studies by Dery et al. it was found that one of the chaperones, the HSP90 protein, is able to bind to the PAS domain of the B factor and stabilize it. Another cellular chaperone, HSP70, recognizes another structural motif of the HIF-1a molecule, the so-called oxygen-dependent degradation domain (ODD) (Dery et al., 2005). It should be noted that the role of these protein-protein interactions is unclear; it is assumed that they are necessary for the stabilization of HIF-1a under normoxic conditions. Under hypoxic conditions, at least one of the chaperones (HSP70) is displaced from the complex with HIF-1a by the ARNT protein, which protects the factor structure from targeted proteolysis during 20–30 min of hypoxia. Thus, HSP70 is able to increase the lifetime of the HIF-1a factor under conditions before and after hypoxia and is required for cells to respond properly to oxygen deprivation. In a general assessment of the results of the study of the metabolism of the nervous tissue, general patterns can be distinguished.

We can conclude that HSP 70 and HIF-1a proteins are inevitable companions of pathobiochemical reactions that develop during ischemic brain damage. Under these conditions, they perform a protective function, which is realized through increased synthesis of antioxidant enzymes, stabilization of oxidatively damaged macromolecules, direct anti-apoptotic and mitoprotective action. Thus, in many recent studies, the important role of HSP70 in the implementation of the mechanisms of metabolic adaptation, neuroplasticity and neuroprotection of brain cells is noted, which allows the presentation of HSP70 as a promising target for neuroprotective drug (Kampinga and Craig, 2010; Kim et al., 2018; Belenichev and Aliyeva, 2019; Belenichev et al., 2022).

## 3. Modern strategy for neuroprotection: potential modulators of the HSP70-dependent endogenous neuroprotection

Modulation of Hsp70 chaperone activity is a promising neuroprotective strategy. We have identified several diverse pharmacological preparations and substances that can potentially modulate the HSP70 protein system.

### 3.1. Tamoxifen

Tamoxifen ((Z)-2-[4-(1,2-Diphenyl-1-butenyl)phenoxy]-N,N-dimethyl-ethanamine citrate) belongs to the group of selective estrogen receptor modulators (SERMs) and, depending on the dose, can exert both agonistic and antagonistic effects. Estrogen receptors (ER) are divided into α and β and belong to the family of nuclear transcription factors. Both types of ER are present in all brain cells: in adreno-, GABA-, cholinergic, and serotonergic neurons, as well as in glia. The amount of ER-β is greater in the cortex and hippocampus, and ER-α in the area of the dentate gyrus. In a state of rest, they do not show biological activity and are in contact with proteins (HSP70, HSP90, AP-1, etc.). As a result of the binding of the ligand to the receptor, the conformation of the latter occurs, followed by the release of proteins from the ER-protein complex, which opens access to binding sites on the receptor for transcription and dimerization correlates (Wakade et al., 2008; Arevalo et al., 2011; Pavlov et al., 2017b). Studies have established the ability of tamoxifen to suppress nNOS, mediated through calmodulin, which reduces the production of NO and the accumulation of its damaging product peroxynitrite (Wakade et al., 2008; Paul and Mahanta, 2014; Pavlov et al., 2017b). In addition, tamoxifen reduces the release of excitotoxic amino acids and acts as a scavenger of free radicals, preventing lipid peroxidation. It has been shown that Tamoxifen and 4-hydroxytamoxifen did not enhance the glutamate-induced increase in intracellular calcium and the activation of NMDA receptors (Cyr et al., 2001; Zhang et al., 2007). In addition, Tamoxifen and 4-hydroxytamoxifen blocked the 17β-estradiol-induced glutamate-dependent increase in intracellular calcium concentration. At the same time, in the culture media of hippocampal neurons, Tamoxifen increased the expression of the anti-apoptotic protein bcl-2, which indicates the neuroprotective effect of the drug. Thus, Tamoxifen and 4-hydroxytamoxifen partially exhibit ER agonist properties in the brain, and in the presence of 17β-estradiol (a “pure” ER agonist) they act as competitive ER antagonists. The obtained research results justify the perspective of studying the modulatory activity of tamoxifen in relation to the HSP70 protein (Pavlov and Belenichev, 2014; Table 1). An important point in the antioxidant effect of Tamoxifen was its positive effect on Mn-superoxide dismutase (MnSOD), the main antioxidant enzyme in mitochondria, which regulates the opening of the mitochondrial pore by inhibiting parasitic reactions (Belenichev et al., 2012).

**TABLE 1:** Pharmacological characteristics of tamoxifen, melatonin, HSF-1, and glutamine.

Drug/Pharmacological group	Dose	Pharmacological effects	References
**Tamoxifen**/Selective estrogen receptor modulator	0.1 μM; 10 μM	*In vitro* in suspension of neurons with addition of DNIS to incubation medium, tamoxifen increases the Hsp70 level and the activity of HSH-enzymes, and decreases in the number of apoptotically changed neurons.	Belenichev et al., 2020
	1 mg/kg	Tamoxifen at this dose is an ER agonist. In a model of acute cerebral ischemia tamoxifen citrate increases the Hsp70 level in the mitochondria and cytosol of the brain, increases the level of GSH, reduces the concentration of markers of oxidative and nitrosative stress.	Pavlov and Belenichev, 2014
	1 mg/kg; 5 mg/kg	Low-dose of tamoxifen up-regulated ER-α36 expression and enhanced neuronal survival, increased neuroprotective effects by modulating activates or suppress ER-α36 in an ovariectomized ischemic stroke model.	Zou et al., 2015
	5 mg/kg	The protective effect of tamoxifen is due to the mitigation of apoptosis, gliosis and inflammation, as well as the normalization of ER levels in CA1, leading to improved cognitive outcomes 24 h after a silent hippocampal infarct.	Finney et al., 2021
**Melatonin**/Neuropeptide	5 mg/kg	In carotid artery occlusion, melatonin increased the cytosolic and mitochondrial HSP70 levels, increased the ATP content in the brain, reduced the level of oxidative stress markers.	Belenichev and Bila, 2019
	5 mg/kg	Melatonin prevents cell death resulting from ischemic brain injury, and that its neuroprotective effects are mediated by the activation of Raf/MEK/ERK/p90RSK cascade.	Koh, 2008
	5 mg/kg; 10 mg/kg	Melatonin plays a protective role against ischemia-reperfusion injury by inhibiting autophagy and activating the PI3K/Akt pro-survival pathway.	Zheng et al., 2014
**HSF-1**/Transcription factor	200 μl/kg	HSF-1 increases the expression of HSP70 and HIF-1a in neurons of the sensorimotor cortex, inhibits neuroapoptosis, activates a compensatory shunt for ATP synthesis, reduces markers of oxidative stress.	Belenichev and Bila, 2019; Belenichev et al., 2020
**Glutamine**/Amino acid	0.3 and 0.5 g/kg/d	Glutamine enhances the HSP70 expression by via the hexosamine biosynthetic pathway, which inhibits the NF-κB pathway.	Raizel and Tirapegui, 2018
	25 mg/kg	Glutamine increases the concentration of HSP70 in the cytosol and mitochondria, leads to a decrease in neuroapoptosis.	Belenichev and Bila, 2019
	0.75 g/kg	Glutamine has a protective effect on cerebral ischemic injury by reducing oxidative stress, inflammatory response, and promoting astrocyte proliferation, accompanied by the upregulation of HSP70.	Luo et al., 2019

### 3.2. Melatonin

Melatonin (N-acetyl-5-methoxytryptamine) is a natural hormone of the pineal gland, and is also synthesized by many extrapineal cells: retina, kidney, intestine, ovary, endometrium, glia, astrocytes, lymphocytes, platelets, etc. The substrate for the synthesis of melatonin is serotonin, which is synthesized from tryptophan. The main function of melatonin is to regulate the organism’s circadian rhythms, therefore the highest concentration of the hormone is observed in the evening hours and leads to a decrease in emotional tension, a decrease in body temperature, and the development of sleep. By reducing the secretion of gonadotropic hormones, thyrotropin, corticotropin, somatotropin and increasing the content of serotonin and GABA, melatonin normalizes circadian rhythms, increases mental and physical performance, and reduces the manifestations of stress reactions (Reiter et al., 2016; Ramos et al., 2017).

Several subtypes of melatonin receptors have been identified in the organism: membrane MTNR/MT and nuclear RZR/ROR. The density of MT1 receptors is greatest on cells of the anterior lobe of the pituitary gland and suprachiasmal nuclei of the hypothalamus, and their activation is associated with the somnogenic effect of melatonin. MT2 receptors are found in large numbers in various structures of the brain, lungs, and retina and are involved in the processes of body synchronization, the formation of emotions, pain, and neurodegeneration (Liu et al., 2016). Activation of nuclear RZR/ROR receptors provides immunomodulatory and antitumor effects of melatonin (Hernandez-Ontiveros et al., 2013; Reiter et al., 2016).

At the moment, melatonin is considered as a universal cytoprotector in pathologies of the brain and cardiovascular system. It has antioxidant, immunomodulatory, anti-ischemic, anti-apoptotic, and neuroregenerative effects (Storr et al., 2002; Koh, 2008; Lochner et al., 2013; Yu et al., 2014; Lamont et al., 2015; Srinivasan et al., 2015; Dwaich et al., 2016; Table 1).

The chemical structure of melatonin allows it to act as a “trap” for free radicals, the hyperproduction of which causes cell damage during ischemic brain damage (Galano et al., 2011; Srinivasan et al., 2015; Vrienda and Reiter, 2015; Ramos et al., 2017). The greatest activity of melatonin is established in relation to the nitric oxide system. By inhibiting nNOS and NO binding, melatonin reduces the level of cytotoxic peroxynitrite and other oxygen intermediates (Storr et al., 2002). The introduction of melatonin increases the activity of antioxidant enzymes: SOD, catalase and glutathione peroxidase (GPO), which reduces the severity of oxidative stress and the amount of damage to brain structures (Fischer et al., 2013; Lochner et al., 2013). *In vivo* experiments on a model of cerebral ischemia established the ability of melatonin to restore memory functions, which is associated with its synaptotropic effect, which is realized due to the restoration of signal transmission in dopaminergic, cholinergic and GABAergic synapses (Beni et al., 2004; Koh, 2008; Hernandez-Ontiveros et al., 2013; Srinivasan et al., 2015; Ramos et al., 2017). The anti-apoptotic properties of melatonin can be associated with increasing expression of apoptosis inhibitor proteins from the Bcl-2 and HSP70 family in nervous tissue, as well as inhibiting the activity of key apoptosis enzymes from the caspase family (Koh, 2008; An et al., 2016). Melatonin increases the expression of HSP70 mRNA, improves the neurological status of rats after carotid occlusion, and promotes the activation of compensatory mitochondrial-cytosolic shunts (Belenichev et al., 2017). Melatonin, due to the induction of the Nrf2 transcription factor, contributes to an increase in the expression of genes of GSH-dependent enzymes, and thus can increase the HSP70 concentration. The prospect of using melatonin as a neuroprotector is also due to its high permeability through the blood–brain barrier and low toxicity (Yu et al., 2014; Srinivasan et al., 2015; Dwaich et al., 2016; Tordjman et al., 2017).

### 3.3. Heat shock factor-1

Heat shock factor-1 (HSF-1) is the main transcriptional regulator of the cellular response to various types of stress conditions (hyperthermia, hypoxia, radiation, heavy metals, etc.) (Neef et al., 2011). In the absence of stress, HSF-1 is in an inactive state as a monomer connected to HSP. Activation of HSF-1 transcriptional activity is a redox-dependent process. As a result of oxidative stress, denatured proteins accumulate, which serves as a signal for the dissociation of the HSF-1/HSP complex (Xing et al., 2004; Mendillo et al., 2012; Tang et al., 2016). The latter, acting as a chaperone, restores damaged polypeptide chains or ensures their proteasomal degradation. In turn, HSF-1 homotrimerizes and is sent to the nucleus, where, connecting to the regulatory heat shock elements (HSE) in the promoter regions of DNA, it starts the process of transcription of Hsp genes. The increased expression of HSPs continues until their numbers are sufficient to block HSF-1 (Nagai et al., 2010).

In addition to the regulation of the HSP system, HSF-1 is involved in the expression of many genes under normal conditions of cell life and regulates the processes of carbohydrate metabolism, RNA splicing, apoptosis, ubiquitinylation and protein degradation, detoxification, transport of small molecules, and intracellular signaling (Neef et al., 2011; Mendillo et al., 2012; Otsuka et al., 2016; Su et al., 2016). The administration of HSF-1 (200 μl/kg) to rats after bilateral carotid artery occlusion contributed to a significant increase in the expression of Hsp70 mRNA, HIF-1α mRNA, and HSP70 concentration, as well as to the inhibition of neuroapoptosis in the sensorimotor cortex and CA1 zone of the hippocampus (Belenichev et al., 2017; Table 1).

In recent works devoted to HSF-1, the ability of HSF-1 to protect neurons through HSP-independent mechanisms was revealed, while HSF-1 does not require prior trimerization to perform its neuroprotective function (Xing et al., 2004; Qu et al., 2018). The obtained data confirm the perspective of further study of the mechanisms of neuroprotective action of HSF-1 (Verma et al., 2014; Liu et al., 2019; Steurer et al., 2022).

### 3.4. Glutamine

Glutamine is one of the most common free amino acids in the organism and is metabolized in almost all tissues. This amino acid is synthesized in organism *de novo* by the enzyme glutamine synthetase (GS), but in conditions of cell damage, an acute deficiency of glutamine occurs, which defines it as a conditionally indispensable amino acid (Curi et al., 2005; Cioccari et al., 2015; Cruzat et al., 2018).

Glutamine is an important participant in metabolic processes, energy production, proliferation, and antioxidant protection. So, glutamine provides nitrogen transport and serves as a carbon source. As a result of hydrolysis, an amino group is released from glutamine, which is used in transamination reactions, including the synthesis of aspartic acid, alanine, and phosphoserine. Glutamine nitrogen is involved in the synthesis of purine and pyrimidine bases, which are necessary for cell proliferation and protein synthesis. As a result of the intracellular oxidation of glutamine, ATP is formed, which is of particular value in conditions of glucose and oxygen deficiency in the brain tissue (Welbourne et al., 2001; Curi et al., 2005; Xi et al., 2011; Wang et al., 2015; Dolgodilina et al., 2016; Raizel and Tirapegui, 2018; Kim et al., 2020b).

Glutamine is a precursor of two neurotransmitters: glutamic acid and gamma-aminobutyric acid, and is also used as a substrate for the synthesis of glutathione (GSH) (Tapieroa et al., 2002; Albrecht et al., 2010; Shen, 2013; Dolgodilina et al., 2016; Hayashi, 2018). In turn, GSH supports the intracellular redox potential due to the regulation of redox-dependent signaling and the activity of transcription factors, and also, due to its antioxidant activity, it stabilizes membranes and increases the transmembrane potential of cells (Wischmeyer, 2003; Roth, 2008; Zabot et al., 2014; McRae, 2017; Brovedan et al., 2018). In many research papers, attention is drawn to the close relationship between the concentration of glutathione and the activity of the chaperone system, primarily HSP70 (Leite et al., 2016; Liu et al., 2018; Luo et al., 2019). In view of the above, the study of the mechanisms of influence of glutamine as a neuroprotective agent is extremely relevant (Chen et al., 2008; Belenichev et al., 2015a; Madeira et al., 2018; Table 1).

Analyzing information from various research papers, it is possible to conclude that the problem of increasing the effectiveness of treatment of ischemic stroke remains highly relevant. Endogenous neuroprotection is considered the main direction in solving the problem of combating ischemic damage, the mechanisms of which lead to an increase in the stability of brain cells due to the activation of their own adaptation mechanisms. In this light, chaperone proteins HSP70 are of particular interest, which under conditions of ischemic stress limit protein aggregation and denaturation, support enzyme activity, and increase antioxidant potential.

Thus, the analysis of research papers data determines the perspective and expediency of the study of selected drugs and substances with the aim of establishing the mechanisms of neuroprotective action due to the effect on the expression of the HSP70.

In research has established that in response to damage, an endogenous cytoprotection program is launched, one of the main participants of which is HSP70. The function of this protein was initially associated with a chaperone effect, which consists in ensuring the folding of newly synthesized proteins, the refolding of damaged proteins, and the disposal of irreversibly damaged polypeptides. But during the last decade, studies have appeared confirming its direct antiapoptotic effect. There is also information that HSP70 takes part in the work of the HIF-1α. Under the guidance of HIF-1α, in conditions of hypoxia, the synthesis of glycolytic enzymes such as phosphofructokinase, pyruvate kinase, glyceraldehyde-3-phosphate dehydrogenase, phosphoglycerate kinase, etc. is activated, which improve energy production in conditions of oxygen deficiency. On the other hand, numerous authors speak of a close relationship between the level of HSP70 and the activity of the antioxidant TDS. Therefore, the positive modulation of the HSP70 system is a promising concept of neuroprotection that can improve the effectiveness of the treatment of cerebral strokes. The obtained results confirm the ability of the studied drugs to activate the system of endogenous neuroprotection, namely, the expression of the genes of the HIF adaptation system. By increasing the level of chaperone 70 protein, the studied drugs support the vital activity of the HIF-1α subunit and activate the synthesis of the HIF protein, which increases the expression of adaptation factors, such as: erythropoietin, vascular endothelial growth factor, glycolysis enzymes (lactate dehydrogenase, pyruvate dehydrogenase, phosphofructokinase, aldolase, etc.), and also ensures the operation of alternative energy systems in conditions of hypoxia (malate-aspartate shuttle mechanism).

The high efficiency of HSF-1 can be explained by the fact that it is a natural stress-inducible transcription factor of the HSP family. Under the conditions of accumulation of products of oxidative and nitrosative stress, trimerization of HSF-1 occurs with detachment from the latter of inactivated HSP70 molecules. The activated HSF-1 complex is transported to the nucleus and, connecting with HSE, starts the transcription process of HSP70 genes.

Regarding Tamoxifen, we assume that it interacts with estrogen receptors, resulting in detachment from the last molecules of HSP70, which subsequently acts as a cytoprotector. At the same time, the level of HSP70 mRNA expression under the influence of tamoxifen also has positive dynamics, which indicates the direct participation of the drug in the signaling and transcription of HSP70 mRNA according to the principle of feedback. Activation of SERM β-ER in the brain causes detachment from the last HSP70 proteins, which ensures the entry of these proteins into the cell and the implementation of their biological function. The mechanism of this interaction is associated with the role of HSP70 in maintaining inactive ER that are not associated with estrogens (Zhang et al., 2007; Pavlov and Belenichev, 2014; Cruzat et al., 2018; Belenichev and Bila, 2019). It is assumed that the feedback of the receptor with the steroid ligand leads to conformational changes in the receptor molecule, its release from the complex with HSPs and their entry into the cell. Also, some researchers have put forward a hypothesis about the ability of SERM to enhance the expression of HSP70 due to a stimulating effect on the protein transcription factor – HSF.

The increase of HSP70 under the influence of melatonin is explained by its effect on nuclear transcription processes. It is known that melatonin interacts with MT1 and MT2 receptors, prevents the translocation of the NF-κB factor into the nucleus, suppresses the expression of iNOS, and reduces the level of proinflammatory cytokines TNF-α, IL-1β, IL-6, etc. In addition, melatonin exhibits a powerful antioxidant effect, reduces the amount of free radicals and, as a result, prevents total DNA damage. Since glutamine acts as a precursor of glutathione synthesis, it is possible to assume that the mechanism of its action lies in the restoration of the redox ratio of GSH/GSSG forms of glutathione, which triggers the expression of adaptation genes and prevents damage to proteins, including HSP70 (Guo et al., 2007; Szyller and Bil-Lula, 2021). It is also known that glutathione directly regulates HSP70 activity (Yang et al., 2020; Zhang et al., 2022). Reduced glutathione inhibits lipid peroxidation reactions through chemical interaction and neutralization of free radicals: singlet oxygen, superoxide radical, peroxynitrite, etc.

Thus, it was established that HSF-1, melatonin, tamoxifen, and glutamine have a significant neuroprotective effect in the case of AVCC, which consists in limiting glutamate excitotoxicity, inhibiting oxidative and nitrosative stress, improving energy metabolism, HSP70 and HIF, and restoring the work of TDS. The mechanism of the neuroprotective action of the drugs is due to HSP70-mediated modulation of endogenous neuroprotection mechanisms, which leads to an increase in the level of reduced glutathione and activation of the HIF adaptation system, which increase the resistance of neurons to ischemia (Belenichev et al., 2017).

## 4. Possible pathways for activation of HSP70-dependent mechanisms of endogenous neuroprotection by modulators of the thiol-disulfide system

A number of authors indicate that an increase in the level of HSP70 leads to the normalization of the glutathione link of the TDS and an increase in the resistance of cells to ischemia (Wang et al., 2012; Pavlov et al., 2017a; Santiago and Morano, 2022). The introduction of exogenous HSP70 leads to an increase in the functional activity of the glutathione system in cortical neurons of the ischemic brain of rats. That is, it was shown that HSP70, proteins with pronounced neuroprotective properties, in conditions of ischemia mobilize antioxidant resources in neurons, in particular, increase the level of both cytosolic and mitochondrial glutathione, which prevents the development of oxidative stress (Pavlov et al., 2017a). In addition, it is known that by modulating the level of endogenous reduced glutathione, it is possible to regulate the expression of HSPs in the cell (Belenichev, 2013; Verma et al., 2014). Confirmation of the perspective of using modulators of the TDS system as means of neuroprotection, which implements its mechanism mediated by increasing the level of endogenous neuroprotection factors, is also confirmed by the following. It was established that high levels of reduced glutathione reduce the activity of 20S and 26S proteasomes of the proteolytic pathway of protein degradation, thereby stabilizing the HIF-1α protein. Thus, these data confirm that depletion of the level of RG (reduced glutathione) under hypoxic conditions plays a decisive role in reducing the level of the HIF-1α protein, and conversely, a high level of RG stabilizes the HIF-1α protein in ischemic conditions (Guo et al., 2007; Wang et al., 2012; Sousa Fialho et al., 2021). The importance of glutathione adducts (S-glutathionylation reaction products) in cell signaling and promotion of ROS-induced revascularization in ischemia through increased expression of HIF-1α is noteworthy (Watanabe et al., 2016b). Modern scientific literature reports on attempts to pharmacologically activate HIF-1α (Ayrapetov et al., 2011; McRae, 2017; Li et al., 2020). Activators of HIF-1α can be divided into two groups: activators of transcription and translation of HIF-1α (gene therapy using viral vectors); and inhibitors of degradation and inactivation of HIF-1α [deferoxamine, cobalt dichloride, dimethyloxalylglycine (DMOG), etc.], however, all these studies are currently experimental in nature. New knowledge in the mechanisms of stimulation or suppression of HIF-1α in conditions of hypoxia (ischemia) will allow to expand the possibilities of using the factor as a specific target for pharmacological correction in oncological, cardiovascular, and neurodegenerative diseases (Zhang et al., 2011; Bouhamida et al., 2022; Chen et al., 2022).

We developed the concept of neuroprotection in the acute period of cerebral stroke, which consists in the pharmacological modulation of the level of glutathione – an important participant in the system of endogenous neuroprotection and neuroplasticity and the increase of glutathione – dependent mechanisms of endogenous neuroprotection. As promising modulators of the glutathione link of the TDS of the brain, we selected: selenase – an activator of GPO, glutoxim – an activator of IL-2 expression and *de novo* reduced glutathione synthesis, glutaredoxin – an important cytosolic protein of disulfide-thiol exchange, a regulator of the activity of transcription factors (NF-1, AP-1, and NF-κB), ASK-1 inhibitor of the apoptosis kinase pathway Glutaredoxin (GRx-1).

### 4.1. Glutaredoxin

Glutaredoxin is one of the most important enzymes in the processes of disulfide reduction and deglutathionylation. Under conditions of oxidative stress, Grx participates in the S-glutathionylation process, while when oxidative stress is reduced, it catalyzes the deglutathionylation reaction (Lu, 2013; Lu and Holmgren, 2014). Glutaredoxin carries out GSH-dependent reduction in proteins of oxidized cysteine residues (Cys – SOH, Cys – SO2H), mediated and intermolecular disulfide bonds (-S-S-) to the redox-active thiol state (Ren et al., 2017). Thiol-containing proteins are widespread in cells. These include enzymes of energy metabolism (phosphofructokinase, glyceraldehyde-3-phosphate dehydrogenase, α-ketoglutarate dehydrogenase, and I complex of respiratory enzymes), enzymes of signaling cascades (protein kinase A, protein kinase Cα, protein tyrosine phosphatase 1b, and protein phosphatase 2A), transcription factors (NF-κB, HIF1, and AP1), and many others. The oxidative shift in the redox potential of cells leads to the formation of disulfide bonds both within protein molecules and between proteins, as well as between protein and glutathione. The formation of mixed glutathione-protein disulfides (S-glutathionylation) is considered as a redox regulatory mechanism that modulates the activity of thiol proteins and related metabolic pathways, signal transduction, and gene expression (Lu and Holmgren, 2014). However, long-term accumulation of oxidized and glutathionylated proteins is associated with the activation of apoptotic pathways in the cell. In the Grx system, electrons are transferred from NADPH to glutathione reductase, then to oxidized glutathione with the formation of RG, which, in turn, reduces oxidized glutaredoxin. Substrates for Grx are disulfides and mixed disulfides (Brigelius-Flohé and Maiorino, 2013; Fisher, 2017). The reduction of disulfides catalyzed by Grx can proceed in two ways, monothiol and dithiol, with the participation of one or two Cys residues in the active site, respectively. The monothiol mechanism leading to deglutathionylation is perhaps the most general function of glutaredoxin. Glutaredoxin is an electron donor for ribonucleotide reductase, plays a role in cell differentiation/proliferation, has anti-apoptotic functions, along with the ability to reduce dihydroascorbate to ascorbate. All these reactions proceed according to the monothiol mechanism. Glutaredoxin plays an important role in the protection of cells from apoptosis using various mechanisms. For example, the value of glutaredoxin in regulating the redox status of the serine/threonine kinase Akt has been described through a GSH-dependent mechanism by dephosphorylation and inactivation of Akt. The inhibitory ability of glutaredoxin on ASK1 kinase activity is described. Glutaredoxin has an activating effect on transcription factors (Watanabe et al., 2016a; Ryu et al., 2018; Table 2).

**TABLE 2:** Pharmacological characteristics of glutaredoxin, glutoxim, sodium selenite, and angiolin.

Drug	Dose	Pharmacological effects	References
**Glutaredoxin**/Redox enzyme	0.5–1.5 μM	Transduced glutaredoxin 1 protein (PEP-1-GLRX1) increased cell viability under oxidative stress and significantly reduced intracellular reactive oxygen species and levels of DNA damage.	Ryu et al., 2018
	200 mkl/kg	Glutaredoxin enhanced the level of ATP and ADP, and also increased the expression of HSP70 and HIF-1a in the model of acute cerebral ischemia.	Belenichev et al., 2020
**Glutoxim**/Thiopoietin	1, 3, 6, and 100 μg/ml	Glutoxim has a immunomodulating effect on lymphocytes showing chemoattractant activity on cells and inhibiting fMLP-induced chemotaxis.	Fimiani et al., 2002
	50 mg/kg	Glutoxim increased the receptor-mediated expression of GR, GPx and GTS, increased the expression of HSP70 in the model of acute cerebral ischemia.	Belenichev et al., 2020
	60 mg/day	The use of glutoxim in patients with sepsis and septic encephalopathy did not affect mortality, but reduced the symptoms of post-septic syndrome	Kobeliatsky et al., 2021
**Sodium selenite** Micronutrient	50 μg/kg	Selenase activates selenium-dependent GPO, demonstrates antioxidant effect, limits the expression of iNOS and the production of cytotoxic NO derivatives.	Belenichev et al., 2017
	0.2 mg/kg	Selenium pre-treatment within the physiological dosage attenuates glutamate toxicity and hypoxia-induced cell damage *in vitro* and ameliorates ischemic brain injury *in vivo*.	Mehta et al., 2012
	0.3 mg/kg	Se increases LP, GSH, vitamin E, Ca levels, and Ca2+-ATPase activities on pentylenetetrazole-induced brain injury.	Pillai et al., 2014
**Angiolin**/Antiischemic, antioxidant	50–100 mg/kg	Angiolin, increases the expression of HSP70 and HIF-1a, improves the ultrastructure of neuronal mitochondria, inhibits neuroapoptosis, increases the GSH concentration and the GSH enzymes activity in various models of cerebral ischemia.	Kucherenko et al., 2018b; Belenichev et al., 2022
	50 mg/kg	Angiolin has anti-ischemic and endothelial protective effects.	Chekman et al., 2017

**FIGURE 2:**
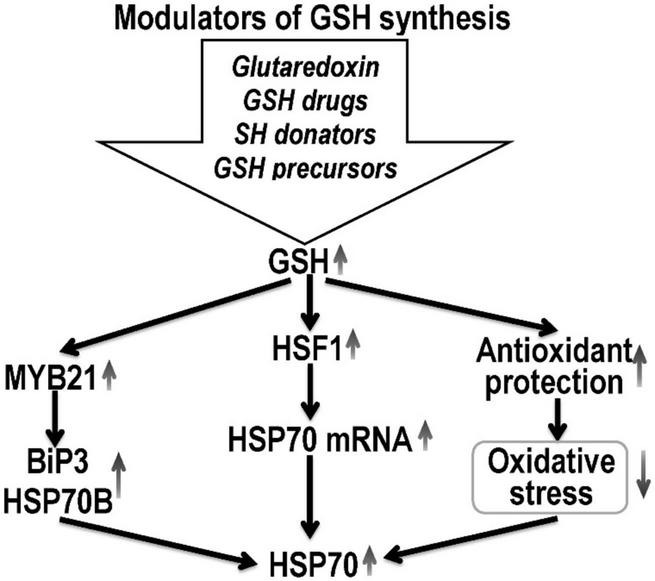
Hypothetical mechanism of HSP70 expression regulation by pharmacological glutathione modulators. Glutathione promotes HSF1 nuclear translocation and enhances the expression of HSP70 mRNA, and subsequently increases HSP70 concentration. GSH can induce HSP expression at the transcript level through the activation of its promoters. Thus, high glutathione concentrations increase the expression of myb21, which activates the promoters of the HSP gene (BiP3 HSP70B) and increase the HSP70 expression. Also, glutathione, by increasing the level of antioxidant protection against oxidative stress, increases the stability of HSP70 mRNA. The possibility of post-translational modification of HSP by S-glutathionylation is also not ruled out.

Thus, glutaredoxin makes a significant contribution to the antioxidant protection of cells against the destructive effects of oxidative stress (Figure 2), which causes the formation of intra- and intermolecular disulfide bonds in proteins, the oxidation of functional SH-groups with the formation of sulfonic acid and the subsequent proteosomal degradation of the protein (Ren et al., 2017).

### 4.2. Glutoxim

According to modern concepts, the glutathione system, namely the GSH/GSSG ratio, is the most important of all known cell redox systems for the regulation of many cellular processes. It is known that a change in the ratio of GSH/GSSG, the redox balance of the cellular environment, is a necessary regulatory mechanism for the activation of a number of transcription factors (AP-1 and NF-κB). Analysis of the literature shows that the majority of works are devoted to the study of the influence of reduced glutathione on various aspects of the cell’s vital activity, the mechanism of action of oxidized glutathione in pathological conditions is still poorly understood (Aquilano et al., 2014). Tissue hypoxia, observed in a number of pathological processes, inevitably leads to their damage. One of the main causes of damage and death of cells in conditions of hypoxia is the disruption of the ion balance maintenance systems. Inhibition of the activity of Na, K-ATPase, the main ion-transporting protein of the plasma membrane of cells, which occurs when oxygen concentration decreases, is considered one of the earliest and critical events for cell viability. In a number of works, the authors note that at the concentrations of oxidized glutathione that exist *in vivo* under oxidative stress and hypoxia, glutathionylation of the SH groups of the α-subunit of Na, K-ATPase occurs, which prevents the oxidation of these SH groups (Ballanyi, 2004). At the same time, the SH groups of Na, K-ATPases, which are important for enzymatic activity, are protected from glutathionylation due to binding to the active center of adenyl nucleotides, primarily ATP. Glutathionylation is eliminated under the action of appropriate enzyme systems (for example, glutaredoxin in the presence of NADP H). Thus, glutathionylation in the state of hypoxia protects the SH-groups of the catalytic subunit of Na, K-ATPase, essential for activity, from irreversible oxidation, while simultaneously reducing the activity of the enzyme, which normally consumes a significant amount of ATP (Ballanyi, 2004).

Glutoxim (glutamyl-cysteinyl-glycine-disodium) belongs to the class of thiopoietins, which modulates intracellular thiol exchange, and is currently used as an immunostimulating agent. Being an analog of endogenous oxidized glutathione, it has a fairly high bioavailability. It has immunocorrective, hemostimulating activities, increases the resistance of cells and the body as a whole in local and generalized chronic infections, increases the effectiveness of therapy in intracellular infections, eliminates the manifestations of a non-specific syndrome of chronic diseases (Dolgodilina et al., 2016). In addition, this drug reproduces the effects of IL-2, with the help of the expression of its receptors, which can lead to a decrease in cytotoxic edema in the acute period of ischemia (Raznatovskaya, 2015). Glutoxim, like GSSG, acts as a substrate for RG and γ-glutamyl transpeptidase (γ-GT), participates in glutathionylation reactions (Townsed et al., 2008). The protection of cellular structures from the toxic effect of free radicals is due to the receptor-mediated increase in the expression of enzymes of the second phase of detoxification of xenobiotics, including glutathione reductase, glutathione peroxidase, glutathione-S-transferase, glucose-6-phosphate dehydrogenase, heme oxygenase-1, an increase in the intracellular level of reduced glutathione, due to the supply plastic material into the cell (structural amino acids) (Townsed and Tew, 2009; Townsed et al., 2009). The positive effects of the drug on LP markers are described, glutoxim reduces the level of diene conjugates and malonaldehyde against the background of an increase in the antioxidant enzymes SOD and catalase (Belenichev et al., 2020; Table 2). Today, the drug is successfully used in phthisiology, oncology, gastroenterology, gynecology, narcology, and toxicology, surgery (Fimiani et al., 2002; Wasinger et al., 2018).

### 4.3. Sodium selenite

**FIGURE 3:**
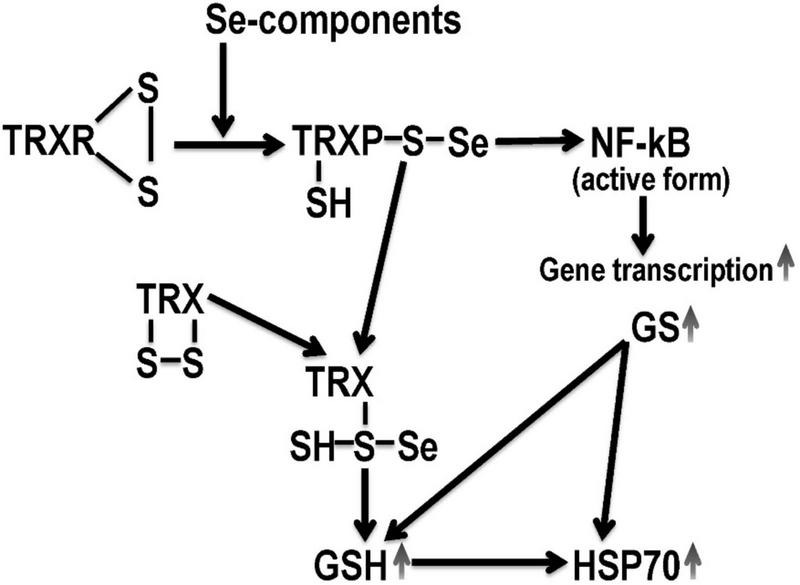
Hypothetical mechanism of selenium derivatives neuroprotection. TRX, thioredoxin; TRXR, thioredoxin reductase; GSH, reduced glutathione; GS, glutathione synthetase; NF-κB, nuclear transcription operator. Selenium derivatives activate GSH-dependent mechanisms of endogenous neuroprotection, which are mediated by an increase in HSP70 expression.

Selenium, which is part of the medicinal product, participates in energetic, metabolic reactions of the body, stimulates the conversion of methionine into cysteine and increases the synthesis of glutathione, which contributes to the overall increase in the antioxidant potential of cells (Huang et al., 2012; Weekly and Harris, 2013; Zoidis et al., 2018). Even in conditions of glutathione deficiency, selenium provides effective antioxidant protection (Tajes et al., 2013). The regulatory functions of selenium reflect the proteins and enzymes, which include a trace element. Selenium is part of GPO and thioredoxin reductase, selenoprotein P (Cardoso et al., 2012; Rahmanto and Davies, 2012). Selenium deficiency leads to a violation of cellular integrity, changes in the metabolism of thyroid hormones, and the activity of biotransforming liver enzymes (Zwolak and Zaporowska, 2012; Bera et al., 2013; Rose and Hoffmann, 2015). *In vivo*, selenoproteins bind to the endothelium of blood vessels and prevent the harmful effects of peroxynitrite. Selenium increases the activity of not only enzymes that contain selenium, but also superoxide dismutase. Selenium exhibits an anti-apoptotic effect by blocking the activation of caspase 3 and DNA fragmentation (Rahmanto and Davies, 2012; Liu et al., 2021). The ratio of reduced and oxidized glutathione is considered as the main redox system that supports the redox homeostasis of the mitochondrial matrix and protects the proteins and DNA of mitochondria from the action of reactive oxygen species. Along with glutathione, protection of cell macromolecules from reactive oxygen species, as mentioned above, is provided by selenium-dependent glutathione peroxidase and thioredoxin reductase (Richie et al., 2014). An important property of thioredoxin reductase is the regulation of redox-sensitive transcription factors. This is due to its ability to restore -SH groups of transcription factors, which are critical for binding to DNA (Pillai et al., 2014; Figure 3). Since all transcription factors have cysteine residues in DNA-binding domains that are highly susceptible to oxidation, thioredoxin is able to maintain them in a restored, functional state. In particular, this was established for such transcription factors as p53 and NF-κB, each of which plays a significant role in the mechanisms of proliferation and apoptosis (Mehta et al., 2012; Albensi, 2019). Thioredoxin forms an inactive complex with the N-terminal fragment of ASK-1 kinase, which regulates the process of apoptosis in the cell (Yu et al., 2014). A number of research papers determine the effectiveness of sodium selenite in myocardial infarction. Selenium compounds have an immunomodulating, radioprotective effect due to the function of glutathione peroxidase, which provides the recovery of hydroperoxides and other products of free radical reactions and regulates the release of lipoxygenase and cyclooxygenase metabolites of arachidonic acid (Koszta et al., 2012; Costa et al., 2014; Lener et al., 2015; Okuyama et al., 2015).

In studies have shown that the modulation of thiol status by the introduction of positive TDS modulators in experimentally justified doses: selenase (sodium selenite) (50 μg/kg), glutoxim (50 mg/kg), and glutaredoxin (200 μl/kg) to animals with AVCC led to less damage of the mitochondrial ultrastructure of neurons in the sensorimotor cortex, to a decrease in the intensity of oxidative and nitrosative stress, to an increase in the concentration of reduced glutathione and an increase in the concentration of mitochondrial and cytosolic HSP70. In animals with carotid artery occlusion treated with these compounds, the concentrations of ATP and ADP in the cytosol and mitochondria of the brain were higher than in the control group (Belenichev et al., 2017; Table 2). Thus, the conducted experimental studies *in vitro* and *in vivo* established that positive modulation of the state of the TDS was manifested in a complex neuroprotective effect, which was provided by antioxidant, energy-modulating, mitoprotective action, as well as the ability to influence subtle mechanisms of endogenous neuroprotection and compensatory-adaptive reactions, aimed at the modulating action of HSP70 proteins. The combination of antioxidant properties and the ability to modulate gene transcription in the studied drugs leads to a more pronounced effect on the expression and synthesis of the studied protective proteins. The analysis of the obtained data showed that the possible mechanisms of the neuroprotective effect of the studied drugs in the conditions of cerebral ischemia are as follows: Selenase – activates selenium-dependent GPO; serves as a “trap” of free radicals, thereby realizing its ability to reduce the level of peroxide compounds; limits the expression of iNOS and the production of cytotoxic NO derivatives. Glutoxim – protects mitochondrial membranes from the harmful effects of free radicals in the S-glutathionylation reaction; serves as a substrate for GR, γ-GT (in the synthesis of *de novo* reduced glutathione); suppresses the expression of pro-inflammatory interleukins – IL-1β, TNF-α, TNF-α – dependent expression of iNOS and thus indirectly prevents the formation of mitochondrial dysfunction (Kobeliatsky et al., 2021). Glutaredoxin makes a significant contribution to the regulation of signal transduction through the ratio of glutathionylation and deglutathionylation processes; is incorporated into the C-terminal segment of ASK-1, thereby inhibiting ASK-1 kinase activity and the subsequent signal transmission pathway (Kekulandara et al., 2018).

Summarizing the obtained data, it should be emphasized that the restoration of the thiol status of the neuron manifests itself in a pronounced neuroprotective activity, which consists in the prevention of cell death, reduction of neurological deficits and the death of experimental animals.

### 4.4. L-lysine 3-methyl-1,2,4-triazolyl-5-thioacetate

**FIGURE 4:**
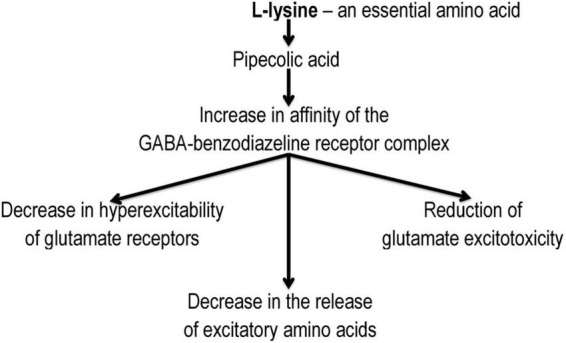
Mechanism of L-lysine action in cells damaged by ischemia/hypoxia.

Angiolin (L-lysine 3-methyl-1,2,4-triazolyl-5-thioacetate) exhibits anti-ischemic, antioxidant properties. It is known that L-lysine can be transformed in the body into pipecolic acid, which increases the affinity of GABA-benzodiazepines-receptor complex and reduces glutamate excitotoxicity (Belenichev et al., 2015b; Nagornaya et al., 2015; Kucherenko et al., 2018a; Figure 4). The mechanism of this action of Angiolin may be associated with its effect on the level of HSP70. Angiolin at a course administration (50–100 mg/kg) to Mughal gerbils with irreversible occlusion of the common carotid artery and Wistar white rats with occlusion of the middle cerebral artery reduced the intensity of nitrosative stress in the ischemic brain (decrease in the level of nitrotyrosine) and increased the expression of endogenous neuroprotection agents – HSP70 in the cytosol and mitochondria of neurocytes (Table 2). Angiolin increases the survival of CA-1 neurons in the hippocampus zone, activates the expression of bcl-2, inhibits the formation of nitrotyrosine, carbonated proteins, increases the content of ATP, malate and pyruvate, an inhibitory transmitter GABA has a beneficial effect on behavioral responses, on the manifestations of neurological symptoms in animals with chronic alcohol intoxication (P McGraw scale). It has been established that the Angiolin protective effect is aimed at normalizing the thiol-disulfide balance of neurons under ischemia conditions – increasing the activity of glutathione-dependent enzymes against the background of an increase in the content of reduced thiol intermediates and a decrease in their oxidized forms (Belenichev et al., 2022). The course administration of Angiolin to animals with chronic cerebral ischemia leads to a decrease in mitochondria with signs of ultrastructural disorders in the CA1 zone of the hippocampus, which may indicate a mitoprotective link in the neuroprotective mechanism of action of the drug.

The mechanism of this action is due to the ability of the L-lysine metabolite – pipecolic acid to limit transmitter autokoidosis and due to the properties of Angiolin to activate compensatory mechanisms, to inactivate cytotoxic forms of NO, to inhibit the NO-dependent mechanism of neuroapoptosis and to increase the bioavailability of this transmitter (Chekman et al., 2017). Also, due to its antioxidant properties, Angiolin is able to influence ROS- and SH-SS-dependent mechanisms of Red/Oxi regulation and transcription, which can lead to an increase in HSP70 expression.

## 5. Conclusion

Currently, the role of endogenous neuroprotection and its intermediate, HSP70, in the regulation of the mechanisms of neuron survival under conditions of cerebral ischemia is being considered. HSP70 performs a chaperone function and is involved in the regulation of growth, development, transfer of genetic material, signaling, and cell death. The neuroprotective properties of HSP70 have been confirmed in various models of ischemic injury *in vitro* and *in vivo*. The neuroprotective effect of HSP70 under conditions of ischemia is explained by its direct anti-apoptotic, antioxidant activity and influence on the TDS of the brain. The works devoted to the study of HSP70 under conditions of cerebral ischemia show its positive effect on the energy metabolism of the brain, which is implemented through a direct mitoprotective effect, as well as through an increase in ATP production in compensatory cytosolic-mitochondrial shunts (Turturici et al., 2011; Pavlov et al., 2017a; Andréasson et al., 2019; Belenichev and Bila, 2019). HSP70 stabilizes and prolongs the HIF-1α “lifetime,” which in turn activates and regulates the malate-aspartate shuttle mechanism. It has been established that the HSP70 expression level in the brain determines the severity of neurological disorders in the modeling of stroke in laboratory animals.

We put forward a hypothesis that an increase in endogenous HSP70 through the use of pharmacological agents in ischemic neurodestruction may be a new direction in neuroprotection and treatment of cerebral strokes. The above-described properties of chaperone proteins in cellular reactions during cerebral ischemia raise the question of the development of new drugs capable of modulation/protection of genes encoding the synthesis of HSP 70 and HIF-1a proteins. Strategies aimed at pharmacological modulation of HSP70 require the maintenance and increase in HSP70 expression, thereby preventing progression toward a more severe course of a stroke and allowing to the mortality reduction. Thus, HSP70 is attractive as a therapeutic target in the treatment of cerebral strokes. The development of new approaches to neuroprotection by creating agents – positive HSP70 modulators is an important task of modern pharmacology. In this respect, drugs with a high neuroprotective potential – tamoxifen, melatonin, glutamine, HSF-1, as well as pharmacological agents that increase the expression of HSP70 by acting on the TDS – selenium compounds, glutaredoxin, glutoxim, and Angiolin are of particular interest.

The established mechanisms of action of HSP70 modulators can contribute to the creation of a new generation of effective drugs that have a targeted effect on the key target links of endogenous neuroprotection mechanisms in conditions of acute cerebrovascular disorders.

## Data availability statement

The original contributions presented in this study are included in the article/supplementary material, further inquiries can be directed to the corresponding author.

## Author contributions

IB designed the research. IB and OA wrote the original manuscript. OP and NB performed the literature search and revised the manuscript. OA made the illustrations. All authors contributed to the article and approved the submitted version.
